# No temporal trends in the prevalence of atypical scrapie in British sheep, 2002–2006

**DOI:** 10.1186/1746-6148-4-13

**Published:** 2008-04-02

**Authors:** K Marie McIntyre, Victor J del Rio Vilas, Simon Gubbins

**Affiliations:** 1 Institute for Animal Health, Pirbright Laboratory, Ash Road, Pirbright, Surrey GU24 0NF, UK; 2 Centre for Epidemiology and Risk Analysis, Veterinary Laboratories Agency, Weybridge, New Haw, Addlestone, Surrey KT15 3NB, UK

## Abstract

**Background:**

So-called atypical scrapie was first identified in Great Britain (GB) in 2002 following the introduction of wide-scale scrapie surveillance. In particular, abattoir and fallen stock surveys have been carried out in GB since 2002, with a total of 147 atypical positives identified by the end of 2006. The results of these surveys provide data with which to assess temporal trends in the prevalence of atypical scrapie in sheep in Great Britain between 2002 and 2006.

**Results:**

Using the results of abattoir and fallen stock surveys, the prevalence of atypical scrapie (percentage of samples positive) was estimated. The prevalence in the abattoir and fallen stock surveys, for all years combined, was 0.09% (95% confidence interval (CI): 0.08%–0.11%) and 0.07% (95% CI: 0.05%–0.11%), respectively. There were no significant temporal trends in either survey. Comparing the surveys' results, there were no significant differences in annual prevalence or the prevalence within *PrP *genotypes. For the abattoir survey, the *PrP *genotype with the highest prevalence was AHQ/AHQ, which was significantly higher than all other genotypes, except ARR/AHQ, AHQ/ARH and ARH/ARQ.

**Conclusion:**

The estimated prevalence of atypical scrapie was similar in both the abattoir and fallen stock surveys. Our results indicate there was no significant temporal trend in prevalence, adding to evidence that this atypical form of scrapie may be a sporadic condition or, if it is infectious, that the force of infection is very low.

## Background

Following the introduction of wide scale scrapie surveillance throughout the European Union in 2002, a number of anomalous positive results were identified in abattoir surveys in several countries [1-4]. At the same time, a novel strain of scrapie, Nor98, was identified, initially in Norway [5] and, subsequently, in other countries [6-9]. Several features were recognized as being common between the unusual abattoir results and Nor98, and both are now classified as atypical scrapie, which is distinct from the classical form of disease [10]. Retrospective analysis of samples has since identified atypical scrapie in a sheep from 1989 [11], suggesting atypical scrapie may have been present in sheep for many years.

Although a strong genetic predisposition at the *PrP *gene affecting susceptibility to classical scrapie has been identified [12-14], such patterns are not as obvious for atypical scrapie. However, associations have been found between atypical scrapie and animals carrying certain alleles, for example, ARR and AHQ [15-18], both of which are generally associated with resistance to or a longer incubation period of classical disease. A further polymorphism at codon 141 (substitution of leucine (L) to phenylalanine (F), in the ARQ haplotype) has also been linked with increased susceptibility to atypical disease [15-18].

Data on atypical scrapie from abattoir and fallen stock surveillance in GB, after five years of active surveillance, allows the investigation of patterns in the detectable prevalence of infection within the sampled populations. In particular, if temporal trends are evident from the results, then they may suggest ongoing transmission. Such trends could be a reflection of two things: either, changes in the national flock genotype profile, the denominator, as a result of selective breeding programmes under the National Scrapie Plan for Great Britain (NSP) [19], or changes in the number of cases, the numerator, as a result of transmission. This assumes that all things would be kept equal from a methodological point of view. Variations in the prevalence could also be a result of changes in the sampling programme or due to the application of tests with different sensitivities at different points in time. For example, Tongue and others have reported the increased sensitivity of the BioRad ELISA versus that of the Prionics Check Western blot test [20]. The latter was used in both surveys at the beginning of 2002. Temporal changes in the prevalence between genotypes are likely to reflect specific genotypic differences affecting susceptibility to disease. Further to this, if the prevalence of atypical scrapie differs between the two surveys, then this may allow comparison of risk factors within their respective target populations.

Within this research we investigated temporal trends in the occurrence of atypical scrapie using the results of the abattoir and fallen stock surveys, initially examining the overall prevalence, but then stratifying by sampling year and the three main codons (136, 154 and 171) on the *PrP *gene associated with classical scrapie. Comparison of the prevalence of atypical scrapie between the surveys was also undertaken.

## Methods

**Figure 1:**
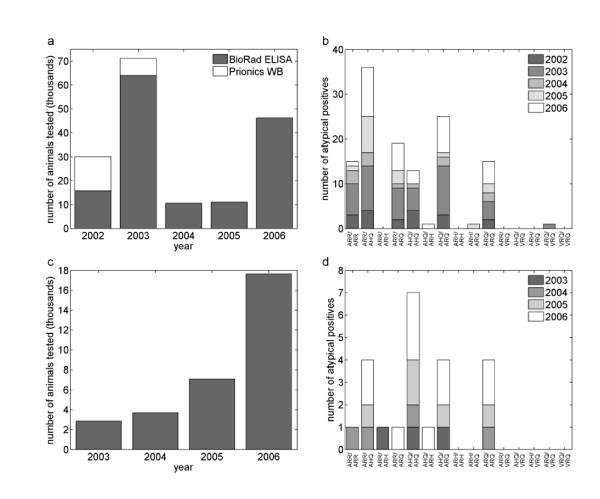
**Surveillance data on atypical scrapie in Great Britain (GB)**. (*a*) Number of animals tested within the abattoir surveys by year and diagnostic test. (*b*) *PrP *genotype distribution of samples which were positive for atypical scrapie each year in the abattoir surveys. (*c*) Number of animals found dead on farm tested between 2003 and 2006. (*d*) *PrP *genotype distribution of samples which were positive for atypical scrapie each year in the fallen stock surveys.

The scope, samples and data collected in the GB abattoir and fallen stock surveys have previously been described elsewhere [2,21,22]. The results of the abattoir surveys were used to provide the number and *PrP *genotype of samples positive for atypical scrapie, and the number of animals tested each year between 2002 and 2006 (Figures 1a, b). Two diagnostic tests were used on samples taken in 2002: the Bio-Rad Platelia ELISA [23], and the Prionics Check Western blot (WB; [24]); in subsequent years only the Bio-Rad ELISA was used (Figure 1a). Importantly, it is not clear whether the Prionics WB was able to detect atypical scrapie [10], and the results for this test were not utilised within the study.

The *PrP *genotypes of animals negative for atypical scrapie were calculated according to the availability of data for each year, for those animals of known genotypes. All animals were genotyped in 2002, though there were around 1000 animals for which this information was missing. The genotype distribution for these animals was assumed to be the same as for those with known genotypes. All animals sampled between January and March 2003 were genotyped, and samples for the remainder of the year were assumed to have the same genotype distribution. Only positive samples were genotyped after 2003 and, hence, the genotypes of the animals sampled for 2004, 2005 and 2006 were estimated from the population structure of the national flock based on data from the NSP [see Additional file 1: table 1, for the population structure].

The results of the fallen stock surveys were used to provide the number and *PrP *genotype of samples positive for atypical scrapie (Figure 1c, d), and the number of animals tested each year between 2003 and 2006. The *PrP *genotypes of animals sampled in the fallen stock surveys are only known for positive samples. The genotype distribution for negative samples in each year was estimated from the population structure of the national flock based on data from the NSP [see Additional file 1: Table 2, for the population structure]. All animals were tested using the Bio-Rad ELISA. Data from a survey in 2002 were not included in the analyses, because of the small sample size (913 animals) and test used (Prionics WB, which would not detect atypical scrapie).

The prevalence of atypical scrapie was estimated as number of positive samples divided by the number tested, with 95% confidence intervals (CI) for the prevalence calculated using the Wilson score interval [25,26]. Differences within and between the abattoir and fallen stock surveys in the overall and annual prevalence were assessed using chi-squared or Fisher exact tests. Further analyses used generalised linear models (GLM) with binomial errors and logit link functions to assess different aspects of the surveillance results. The first model was used to assess temporal trends in prevalence, and whether these differed between surveys (i.e. the initial model included year and survey with an interaction between them). The second was used to assess differences in prevalence amongst *PrP *genotypes and whether these differed between surveys (i.e. the initial model included *PrP *genotype and survey with an interaction between them). The third and final model was used to assess whether there were temporal trends in prevalence in different *PrP *genotypes (i.e. the initial model included year and *PrP *genotype with an interaction between them). As no atypical positives have been identified for certain *PrP *genotypes, this model was examined both with and without the inclusion of these *PrP *genotypes to check they did not influence the potential effects of year as an explanatory variable. This analysis used quasi-binomial errors to correct over-dispersion in the data, and was undertaken only for the abattoir survey; equivalent analysis using the fallen stock data was not undertaken because there were too few positive samples. All models were constructed using backward stepwise deletion of insignificant terms (*P *> 0.05).

## Results

**Figure 2:**
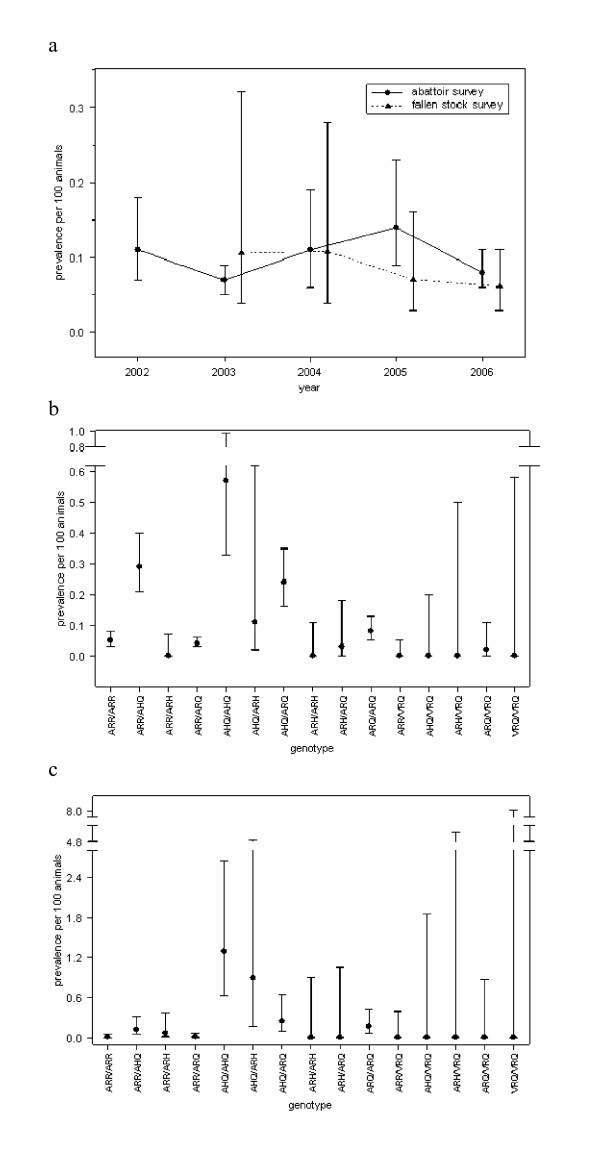
**Estimated prevalence of atypical scrapie in GB per 100 animals tested as part of the abattoir and fallen stock surveys, between 2002 and 2006, or 2003 and 2006, respectively**. (*a*) by year; (*b*) by *PrP *genotype in the abattoir surveys; and (*c*) by *PrP *genotype in the fallen stock surveys. Symbols show the estimates, and error bars the 95% confidence limits calculated using the Wilson score interval.

The estimated prevalence of atypical scrapie from the results of the abattoir and fallen stock surveys for all years combined were 0.09% (95% confidence interval (CI): 0.08%–0.11%) and 0.07% (95% CI: 0.05%–0.11%), respectively. These results were not significantly different from each other (*P *= 0.59), nor were there any differences in the annual prevalence between the abattoir and fallen stock surveys (2003, *P *= 0.46; 2004, *P *= 0.99; 2005, *P *= 0.23; and 2006, *P *= 0.69) (Figure 2a).

There was no significant temporal trend in the prevalence of atypical scrapie (*P *= 0.69), nor any difference in prevalence between the two surveys (*P *= 0.51) (Figure 2a). Furthermore, there were no significant differences in prevalence estimates within *PrP *genotypes for the two surveys (*P *= 0.46; Figure 2b and 2c).

**Figure 3:**
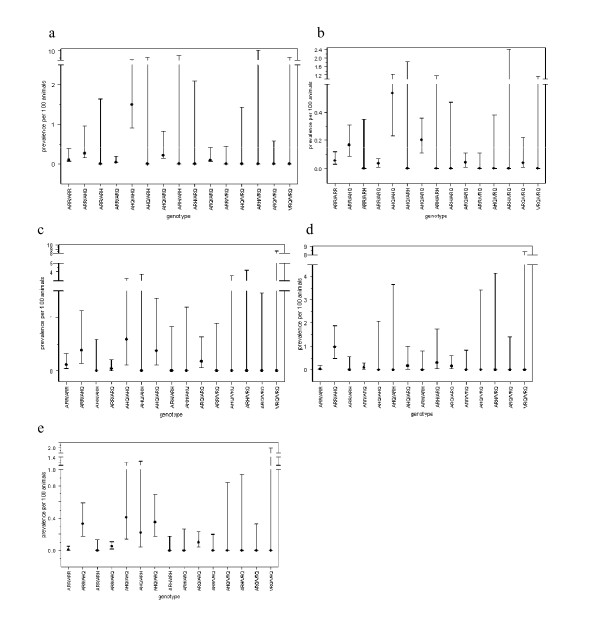
**Estimated prevalence of atypical scrapie per 100 animals tested as part of the abattoir survey for each year stratified by *PrP *genotype**. (*a*) 2002, (*b*) 2003, (*c*) 2004, (*d*) 2005 and (*e*) 2006. Symbols show the estimates, and error bars the 95% confidence limits calculated using the Wilson score interval.

**Figure 4:**
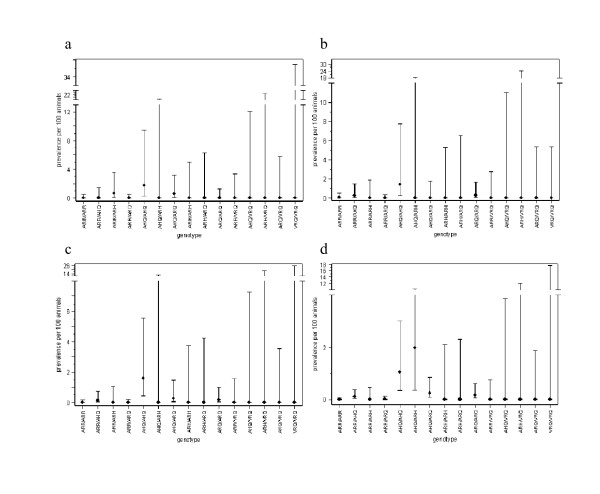
**Estimated prevalence of atypical scrapie per 100 animals tested as part of the fallen stock survey for each year stratified by *PrP *genotype**. (*a*) 2003, (*b*) 2004, (*c*) 2005 and (*d*) 2006. Symbols show the estimates and error bars the 95% confidence limits calculated using the Wilson score interval.

In the generalised linear model (GLM) for the abattoir survey results which included year and *PrP *genotype, there was no significant effect of year (*P *= 0.32), but there was a significant effect of *PrP *genotype (*P *< 0.001), suggesting that the prevalence of atypical scrapie in each genotype did not change over time (Figures 2a and 3). The prevalence in ARR/AHQ, AHQ/ARH and ARH/ARQ was not significantly different from that in AHQ/AHQ (the baseline), the prevalence in AHQ/ARQ was around one half that of AHQ/AHQ, and the prevalence in ARR/ARR, ARR/ARQ, ARQ/ARQ and ARQ/VRQ was around one tenth that of AHQ/AHQ; for the remaining genotypes, no positive samples were found (Table 1; Figures 2b and 3). Although no formal analysis was undertaken, a similar pattern was observed in the fallen stock survey results (Figures 2c and 4; cf. Figures 2b and 3).

**Table 1:** Genotype-specific risk of atypical scrapie being detected in an abattoir survey. (An odds ratio shown in bold is significantly (*P *< 0.05) different from the baseline (AHQ/AHQ).

	Odds ratio		
	
*PrP *genotype	Estimate	95% confidence limits	
		
		Lower	Upper
ARR/ARR	**0.07**	0.03	0.19
ARR/AHQ	0.47	0.21	1.06
ARR/ARH^†^	0	-	-
ARR/ARQ	**0.07**	0.03	0.18
AHQ/AHQ^‡^	1	-	-
AHQ/ARH	0.36	0.03	4.84
AHQ/ARQ	**0.39**	0.17	0.92
ARH/ARH^†^	0	-	-
ARH/ARQ	0.50	0.04	6.70
ARQ/ARQ	**0.13**	0.05	0.33
ARR/VRQ^†^	0	-	-
AHQ/VRQ^†^	0	-	-
ARH/VRQ^†^	0	-	-
ARQ/VRQ	**0.06**	0.00	0.84
VRQ/VRQ^†^	0	-	-

^† ^no positive samples for this genotype.

^‡ ^baseline genotype

## Discussion

In this paper the temporal trends in the occurrence of atypical scrapie were examined, under the premise that if they exist they may provide some evidence for an infectious nature to the disease, through on-going transmission. Further to this, differences in the prevalence of atypical scrapie between abattoir and fallen stock surveys were also examined, as this could lead to comparison of risk factors within their respective target populations.

The prevalence of atypical scrapie was around 0.1% for both the abattoir and fallen stock surveys and, moreover, it did not vary significantly between surveys or years (Figure 2a). This confirms the results of a comparison of scrapie surveillance streams across the EU, using a meta-analysis approach [27]. It is in marked contrast to classical scrapie, where there was large variation in the frequency estimates of scrapie between the abattoir and fallen stock surveys across countries within the EU [28,29].

There were significant differences in the prevalence of atypical scrapie amongst *PrP *genotypes (Figures 2b, c, 3 and 4). The highest prevalence was in the AHQ/AHQ genotype, with other AHQ-bearing genotypes also having a higher prevalence compared with non-AHQ-bearing genotypes. This is similar to previous studies of atypical scrapie, which have also identified an increased risk associated with the AHQ allele [15,17,18,30]. Furthermore, a leucine (L) to phenylalanine (F) substitution at codon 141 of the ARQ haplotype is associated with an increased risk of atypical scrapie [17], but data on this polymorphism were not collected routinely between 2002 and 2006, though they will be in future years [31]. Distinguishing between AL_141_RQ- and AF_141_RQ-bearing genotypes may highlight differences in prevalence in these genotypes, which are not apparent in this study. A high frequency of the AF_141_RQ polymorphism within animals of the ARH/ARQ genotype may also explain the apparent high risk of atypical scrapie in this genotype [17].

In addition, the *PrP *genotypes affected by atypical scrapie are markedly different from those affected by classical scrapie [18,30]. Notably, the atypical positives tend to be detected in animals carrying the ARR or AHQ alleles (Figure 1b, d) while classical positives in GB tend to be detected in animals carrying the VRQ allele. Only the ARQ allele is found in both atypical and classical positives, but differentiating between animals carrying AL_141_RQ and AF_141_RQ may indicate differences here too.

Although the prevalence of atypical scrapie has not changed over time within individual *PrP *genotypes (Figures 3 and 4), the overall prevalence could still change temporally if the frequency of *PrP *genotypes were to change, for example, as a result of selective breeding under the NSP. There has, however, been little effect of the NSP on AHQ: the frequency of this allele in ram lambs genotyped for the NSP has remained approximately constant at around 7% [32]. Moreover, the frequency of the ARQ allele in ram lambs has changed only slightly, decreasing from 29% in 2002 to 22% in 2004 [32]. The relatively small changes in the frequency of alleles associated with atypical scrapie helps explain why the overall prevalence has not changed significantly over time. This is in marked contrast to classical scrapie, where the frequency of the allele associated with the highest risk (VRQ) has decreased from 3.0% in 2002 to 1.7% in 2004 [32] in parallel with a decrease in prevalence.

One potential short-coming of comparisons in the prevalence between abattoir and fallen stock surveys, is that each survey may target a different population or event (infection vs. clinical disease). However, the lack of information on the pre-clinical phase of atypical scrapie makes it difficult to identify precisely how the prevalence may differ between the abattoir and fallen stock populations. For example, the detectable prevalence in each survey could differ if there were differences in the ages or stage of incubation of animals sampled in the two surveys. However, age data are available for the fallen stock surveys, but not the abattoir surveys, making such a comparison impossible. Moreover, stratifying prevalence by age as well as *PrP *genotype will further reduce the number of animals in each age and genotype class and, hence, the statistical power of any comparison. In addition, the high ratio of cases identified through abattoir (126) and fallen stock surveys (23) compared to statutory reporting (six) [8] suggests that the age-at-onset of clinical signs for atypical scrapie is much later than the commercial life-span of a sheep.

It must be remembered that the analyses in this study have required data on the *PrP *genotypes of both positive and negative samples. Although this information is routinely collected for the positive samples, it was only available for negative samples collected in the abattoir surveys between January 2002 and March 2003, and not at all for samples collected in the fallen stock surveys. Consequently, the *PrP *genotypes of negative samples have been inferred from the *PrP *genotype frequencies in the national flock [see Additional file 1]. The estimates for the prevalence of atypical scrapie will clearly depend on the robustness of the inferred frequencies of these genotypes, but this is very difficult to assess. Moreover, any biases in the data used to infer the *PrP *genotypes would result in biases in the risk estimates [14]. The NSP data used were derived from a voluntary ram genotyping scheme in purebred flocks. This scheme requires culling or castration of VRQ-bearing rams [19] and, hence, may underestimate the frequency of these *PrP *genotypes. It is essential that future surveys collect genotype data on at least a proportion of negative samples to allow full use of the surveillance data. This will become more important as the genotype profile of the national flock changes through the impact of the NSP.

## Conclusion

The results of this study indicate that the prevalence of atypical scrapie did not change significantly between 2002 and 2006. Furthermore, it did not differ significantly between the abattoir and fallen stock surveys, which is not the case for classical scrapie. The absence of temporal trends in the prevalence of atypical scrapie adds to evidence that this may be a sporadic condition or, if it is infectious, the force of infection is very low. Recent experimental work has demonstrated that atypical scrapie is transmissible [33], but the evidence for whether or not it is infectious is mixed. Examination of demographic factors and trading patterns has suggested transmission of atypical scrapie could be occurring, albeit slowly [34], suggesting it could be infectious. By contrast, a case-control study of Nor98 in Norway found no risk factors to indicate transmission between flocks [35], suggesting atypical scrapie is sporadic. Ultimately, a combination of evidence from case-control studies, spatio-temporal analysis and laboratory experiments will be necessary to determine whether this disease is infectious or sporadic in nature. A similar approach was utilised in the case of arguments surrounding sporadic- versus variant-Creutzfeldt-Jakob disease [36].

## Authors' contributions

KMM carried out the statistical analyses and drafted the manuscript. VDRV critically revised the manuscript. SG carried out the statistical analysis, critically revised the manuscript and conceived the study. All authors read and approved the final manuscript.

## Supplementary Material

Additional file 1 Table 1. Number of animals tested and number of samples positive for atypical scrapie by *PrP* genotype and year for abattoir surveys in GB, 2002–2006. Table 2. Number of animals tested and number of samples positive for atypical scrapie by *PrP* genotype and year for fallen stock surveys in GB, 2003–2006. 2 MS Excel tables Click here for file

## References

[B1] Buschmann A, Biacabe AG, Ziegler U, Bencsik A, Madec JY, Erhardt G, Lühken G, Baron T, Groschup MH (2004). Atypical scrapie cases in Germany and France are identified by discrepant reaction patterns in BSE rapid tests. J Virol Methods.

[B2] Everest SJ, Thorne L, Barnicle DA, Edwards JC, Elliott H, Jackman R, Hope J (2006). Atypical prion protein in sheep brain collected during the British scrapie-surveillance programme. JGV.

[B3] Madec JY, Simon S, Lezmi S, Bencsik A, Grassi J, Baron T (2004). Abnormal prion protein in genetically resistant sheep from a scrapie-infected flock. JGV.

[B4] Orge L, Galo A, Machado C, Lima C, Ochoa C, Silva J, Ramos M, Pedro Simas J (2004). Identification of putative atypical scrapie in sheep in Portugal. JGV.

[B5] Benestad SL, Sarradin P, Thu J, Schonheit J, Tranulis MA, Bratberg B (2003). Cases of scrapie with unusual features in Norway and designation of a new type, Nor98. Vet Rec.

[B6] Epstein V, Pointing S, Halfacre S (2005). Atypical scrapie in the Falkland Islands. Vet Rec.

[B7] Gavier-Widen D, Stack MJ, Baron T, Balachandran A, Simmons M (2005). Diagnosis of transmissible spongiform encephalopathies in animals: a review. J Vet Diagn Invest.

[B8] Konold T, Davis A, Bone GE, Simmons MM, Kahn J, Blake-Dyke MC, Bracegirdle J, Shimwell CJ (2006). Atypical scrapie cases in the UK. Vet Rec.

[B9] Onnasch H, Gunn HM, Bradshaw JM, Benestad SL, Bassett HF (2004). Two Irish cases of scrapie resembling Nor98. Vet Rec.

[B10] EFSA (2005). Opinion of the scientific panel on biological hazards on classification of atypical transmissible spongiform encephalopathy (TSEs) cases in small ruminants. The EFSA Journal.

[B11] Bruce ME, Nonno R, Foster J, Goldmann W, Di Bari M, Esposito E, Benestad SL, Hunter N, Agrimi U (2007). Nor98-like sheep scrapie in the United Kingdom in 1989. Vet Rec.

[B12] Baylis M, Chihota CM, Stevenson E, Goldmann W, Smith A, Sivam K, Tongue SC, Gravenor MB (2004). Risk of scrapie in British sheep of different prion protein genotype. JGV.

[B13] Dawson M, Hoinville LJ, Hosie BD, Hunter N (1998). Guidance on the use of PrP genotyping as an aid to the control of clinical scrapie. Vet Rec.

[B14] Tongue SC, Pfeiffer DU, Warner R, Elliot H, del Rio Vilas V (2006). Estimation of the relative risk for developing clinical scrapie: the role of prion protein (*PrP*) genotype and selection bias.. Vet Rec.

[B15] EFSA (2006). Report of the working group on the breeding programme for TSE resistance in small ruminants. The EFSA Journal.

[B16] Lühken G, Buschmann A, Groschup MH, Erhardt G (2004). Prion protein allele A(136)H(154)Q(171) is associated with high susceptibility to scrapie in purebred and crossbred German Merinoland sheep. Arch Virol.

[B17] Moum T, Olsaker I, Hopp P, Moldal T, Valheim M, Moum T, Benestad SL (2005). Polymorphisms at codons 141 and 154 in the ovine prion protein gene are associated with scrapie Nor98 cases. JGV.

[B18] Saunders GC, Cawthraw S, Mountjoy SJ, Hope J, Windl O (2006). *PrP* genotypes of atypical scrapie cases in Great Britain. JGV.

[B19] DEFRA (2001). National Scrapie Plan for Great Britain. Schemes Brochure.

[B20] Tongue SC, Wilesmith JW, Nash J, Kossaibati M, Ryan J (2007). The importance of the *PrP* genotype in active surveillance for ovine scrapie. Epidemiol Infect.

[B21] del Rio Vilas VJ, Ryan J, Elliot HG, Tongue SC, Wilesmith JW (2005). Prevalence of scrapie in sheep: results from fallen stock surveys in Great Britain in 2002 and 2003. Vet Rec.

[B22] Elliott H, Gubbins S, Ryan J, Ryder S, Tongue S, Watkins G, Wilesmith J (2005). Prevalence of scrapie in sheep in Great Britain estimated from abattoir surveys during 2002 and 2003. Vet Rec.

[B23] Grassi J, Comoy E, Simon S, Creminon C, Frobert Y, Trapmann S, Schimmel H, Hawkins SAC, Moynagh J, Deslys JR, Wells GAH (2001). Rapid test for the preclinical postmortem diagnosis of BSE in central nervous system tissue. Vet Rec.

[B24] Schaller O, Fatzer R, Stack M, Clark J, Cooley W, Biffiger K, Egli S, Doherr M, Vandevelde M, Heim D, Oesch B, Moser M (1999). Validation of a Western immunoblotting procedure for bovine *PrPSc* detection and its use as a rapid surveillance method for the diagnosis of bovine spongiform encephalopathy (BSE). Acta Neuropathol.

[B25] Agresti A (2002). Categorical data analysis.

[B26] Wilson EB (1927). Probable inference, the law of succession, and statistical inference. J Am Stat Assoc.

[B27] del Rio Vilas VJ, Böhning D, Kuhnert R Evaluating the active surveillance of scrapie in the EU. Advances in Disease Surveillance.

[B28] Anon (2004). Report on the monitoring and testing of ruminants for the presence of transmissible spongiform encephalopathy (TSE) in the EU in 2003, including the result of the survey of prion protein genotypes in sheep breeds.

[B29] del Rio Vilas VJ, Hopp P, Nunes T, Ru G, Sivam K, Ortiz-Pelaez A (2007). Explaining the heterogeneous scrapie surveillance figures across Europe: a meta-analysis approach. BMC Veterinary Research.

[B30] Lühken G, Buschmann A, Brandt H, Eiden M, Groschup MH, Erhardt G (2007). Epidemiological and genetical differences between classical and atypical scrapie cases. Vet Res.

[B31] Anonymous (2007). Commission regulation (EC) No 7727/2007 of 26 June 2007. Official Journal of the European Union.

[B32] Warner RG, Morris D, Dawson M (2006). *PrP* genotype progression in flocks participating in the National Scrapie Plan for Great Britain. Vet Rec.

[B33] Simmons MM, Konold T, Simmon HA, Spencer YI, Lockey R, Spiropoulous J, Everitt S, Clifford D (2007). Experimental transmission of atypical scrapie to sheep. BMC Veterinary Research.

[B34] Green DM, del Rio Vilas VJ, Birch CPD, Johnson J, Kiss IZ, McCarthy ND, Kao RR (2007). Demographic risk factors for classical and atypical scrapie in Great Britain. JGV.

[B35] Hopp P, Omer MK, Heier BT (2006). A case-control study of scrapie Nor98 in Norwegian sheep flocks. JGV.

[B36] Mead S, Mahal SP, Beck J, Campbell T, Farrall M, Fisher E, Collinge J (2001). Sporadic - but not variant - Creutzfeldt-Jakob disease is associated with polymorphisms upstream of PRNP Exon 1. Am J Hum Genet.

