# Supplementary material for: No temporal trends in the prevalence of atypical scrapie in British sheep, 2002–2006
Source: BMC Vet Res. 2008 Apr 2;4:13. doi: 10.1186/1746-6148-4-13 (PMC2397389; doi:10.1186/1746-6148-4-13)
Supplement: Additional file 1 — Table 1. Number of animals tested and number of samples positive for atypical scrapie by PrP genotype and year for abattoir surveys in GB, 2002–2006. Table 2. Number of animals tested and number of samples positive for atypical scrapie by PrP genotype and year for fallen stock surveys in GB, 2003–2006. 2 MS Excel tables [file 1746-6148-4-13-S1.pdf]

Additional file 1. Table 1. Number of animals tested and number of samples positive for atypical scrapie by *PrP* genotype and year for abattoir surveys in GB, 2002-2006.

PrP genotype	2002		2003		2004		2005		2006	
	positive	tested	positive	tested†	positive	tested‡	positive	tested‡	positive	tested‡
ARR/ARR	3	3039	7	12535	3	2741	1	2876	1	11805
ARR/AHQ	4	1497	10	5965	3	780	8	834	11	3284
ARR/ARH	0	230	0	1082	0	652	0	676	0	2874
ARR/ARQ	2	4939	7	20063	1	2773	3	2913	6	12078
AHQ/AHQ	4	267	5	938	1	169	0	181	3	728
AHQ/ARH	0	57	0	207	0	98	0	101	1	448
AHQ/ARQ	3	1423	11	5488	2	534	1	569	8	2298
ARH/ARH	0	47	0	327	0	459	0	469	0	2112
ARH/ARQ	0	179	0	813	0	315	1	324	0	1441
ARQ/ARQ	2	2237	4	9192	2	1152	2	1207	5	5112
ARR/VRQ	0	870	0	3449	0	426	0	446	0	1874
AHQ/VRQ	0	265	0	1005	0	103	0	108	0	454
ARH/VRQ	0	33	0	154	0	88	0	89	0	406
ARQ/VRQ	0	658	1	2588	0	259	0	270	0	1156
VRQ/VRQ	0	57	0	332	0	41	0	42	0	189
total	18	15798	45	64138	12	10590	16	11105	35	46259

† in 2003 negative samples were *PrP* genotyped between January 2003 and March 2003; negative samples tested during the remainder of the year were assumed to have the same genotype distribution.

‡ in 2004-2006 negative samples were not *PrP* genotyped; the genotype distribution for negative samples in each year was estimated from the population structure of the national flock based on data from the NSP.

Table 2. Number of animals tested and number of samples positive for atypical scrapie by *PrP* genotype and year for fallen stock surveys in GB, 2003-2006.

PrP genotype	2003		2004		2005		2006	
	positive	tested†	positive	tested†	positive	tested†	positive	tested†
ARR/ARR	0	758	1	1102	0	2340	0	6587
ARR/AHQ	0	271	1	377	1	757	2	1962
ARR/ARH	1	153	0	200	0	357	0	836
ARR/ARQ	0	760	0	998	0	1934	1	4681
AHQ/AHQ	1	56	1	70	2	127	3	289
AHQ/ARH	0	17	0	17	0	27	1	50
AHQ/ARQ	1	176	0	213	1	381	2	857
ARH/ARH	0	73	0	69	0	99	0	180
ARH/ARQ	0	57	0	55	0	87	0	163
ARQ/ARQ	0	297	1	333	1	564	2	1196
ARR/VRQ	0	111	0	136	0	237	0	506
AHQ/VRQ	0	28	0	31	0	49	0	96
ARH/VRQ	0	13	0	12	0	17	0	28
ARQ/VRQ	0	63	0	68	0	104	0	203
VRQ/VRQ	0	7	0	8	0	11	0	18
total	3	2840	4	3689	5	7091	11	17652

† negative samples were not *PrP* genotyped; the genotype distribution for negative samples in each year was estimated from the population structure of the national flock based on data from the NSP.
